# Executive function deficits and medial temporal lobe atrophy in late-life depression and Alzheimer’s disease: a comparative study

**DOI:** 10.3389/fpsyt.2023.1243894

**Published:** 2023-08-31

**Authors:** Changbiao Chu, Weigang Pan, Yanping Ren, Peixian Mao, Chunlin Yang, Chaomeng Liu, Yi-lang Tang

**Affiliations:** ^1^Innovation Center for Neurological Disorders and Department of Neurology, Xuanwu Hospital, Capital Medical University, National Clinical Research Center for Geriatric Diseases, Beijing, China; ^2^Beijing Key Laboratory of Mental Disorders, National Clinical Research Center for Mental Disorders and National Center for Mental Disorders, Beijing Anding Hospital, Capital Medical University, Beijing, China; ^3^Advanced Innovation Center for Human Brain Protection, Capital Medical University, Beijing, China; ^4^Department of Psychiatry and Behavioral Sciences, Emory University School of Medicine, Atlanta, GA, United States; ^5^Mental Health Service Line, Atlanta VA Medical Center, Decatur, GA, United States

**Keywords:** executive function deficits, medial temporal lobe atrophy, Alzheimer’s disease, late-life depression, receiver operating characteristic

## Abstract

**Objectives:**

Alzheimer’s disease (AD) and late-life depression (LLD) frequently exhibit executive function deficits (EFD) and medial temporal lobe atrophy (MTA) as shared characteristics. The objective of this research was to examine the utility of the Trail Making Test (TMT) and the MTA scale in distinguishing between LLD and AD.

**Methods:**

A study of 100 patients, 50 with AD and 50 with LLD, was conducted using a cross-sectional design. The individuals were subjected to clinical evaluations to assess their level of depression and overall cognitive abilities, which included the Geriatric Depression Scale (GDS), Mini-Mental State Examination (MMSE), and Montreal Cognitive Assessment (MoCA). We evaluated executive function deficits (EFD) through the use of the TMT, which includes both TMT-A and TMT-B. MTA was measured using magnetic resonance imaging. To evaluate the ability of TMT and MTA scale to distinguish between the two groups, a receiver operating characteristic (ROC) curve was utilized. To investigate the connections between MTA and neuropsychological measures, a correlation analysis was performed.

**Results:**

AD patients exhibited notably reduced MMSE, MoCA, and GDS scores, as well as an increased MTA total scores, time spent on TMT-A, and TMT-B compared to LLD patients (*p* < 0.05). TMT-A and TMT-B both exhibited excellent discriminatory power between AD and LLD, achieving area under curve (AUC) values of 92.2 and 94.2%, respectively. In AD patients, there was a negative correlation between MMSE and MoCA scores and MTA scores, while in LLD patients, there was a positive correlation between time spent on TMT-A and GDS scores and MTA scores.

**Conclusion:**

AD patients experience more severe EFD and MTA than LLD patients. The differential diagnosis of AD and LLD can be aided by the useful tool known as TMT. It is important to acknowledge that TMT is capable of capturing only a fraction of the executive function, thus necessitating a cautious interpretation of research findings.

## Introduction

Late-life depression (LLD) is a condition characterized by depressive symptoms that manifest in individuals aged 60 or older, and it was estimated to affect 4–10% of the elderly in the community (1, 2). Unlike major depressive disorder (MDD) seen in younger individuals, LLD is often associated with age-related neurodegeneration, cognitive impairment, and physical complaints (3). On the other hand, Alzheimer’s disease (AD) is a degenerative neurological disorder characterized by progressive and irreversible cognitive dysfunction and behavioral impairment (4). From a histological and anatomical perspective, the presence of extracellular amyloid-β (Aβ) peptide deposition forming “senile plaques” and intracellular tau protein hyperphosphorylation leading to the formation of “neurofibrillary tangles” are considered two prominent features of AD. These pathological hallmarks are significant contributors to the development of dementia and pose challenges in terms of reversibility for affected patients (5). Depressive symptoms are reported in 30–50% of AD patients and the terms “depression” and “dementia” in these patients are heterogeneous with blurred boundaries (6). Previous studies have suggested that depression may be either a risk factor or a consequence of cognitive decline, or that the two conditions may share some common mechanisms (7, 8). Differential diagnosis between LLD and AD is often challenging in clinical practice due to overlap in their symptoms and shared psychopathology (9, 10). Older individuals experiencing LLD are prone to mild cognitive impairment (MCI) compared to depressed individuals under 60 years old, particularly in memory, attention, and executive function. Additionally, individuals with LLD and AD frequently experience similar brain alterations, including microvascular disease, which may play a role in their progression (11).

Misdiagnosis is frequent in distinguishing LLD from AD, with 15–23 percent of AD patients previously diagnosed with depression and about 30% of LLD patients incorrectly diagnosed with AD (12, 13). A correct and timely diagnosis is crucial for starting appropriate treatment, preventing unnecessary diagnostic tests, and developing future interventions for AD. Improvement of depressive symptoms can lead to better cognitive function in young patients with MDD, but executive function deficits (EFD) often persist in LLD groups (14). These deficits affect impulse inhibition, cognitive flexibility, planning and organization, semantic fluency, and selective attention (15). LLD patients with EFD are at a higher risk of developing AD later on Potter et al. (16). Therefore, it is crucial to identify biomarkers of EFD in LLD patients that can differentiate them from AD patients and develop targeted interventions.

White matter hyperintensities are commonly found in both LLD and AD on magnetic resonance imaging (MRI) scans, particularly in subcortical structures and their frontal projections (17, 18). Studies using diffusion tensor imaging have identified microstructural abnormalities in white matter tracts connecting the prefrontal cortex (PFC) with subcortical and posterior cortical regions in LLD, which are associated with executive dysfunction (19, 20). The hippocampus, which is sensitive to altered cortisol, serotonin, and brain-derived neurotrophic factor (BDNF) levels, is also affected by both depression and AD. Additionally, early accumulation of Aβ may lead to hippocampal atrophy, which implies that depression risk may be heightened by AD pathology through hippocampal atrophy (21, 22).

MRI is a radiation-free imaging technique that provides more information compared to computed tomography (CT) and is valuable in identifying hippocampal atrophy in the elderly (23). The Schelten’s scale, also called the medial temporal lobe atrophy (MTA) score, is frequently used in clinical settings to assess the shrinkage of the hippocampus (24). The evaluation of MTA through MRI is commonly included in the routine assessment of individuals experiencing cognitive decline. Various (semi-) automated segmentation tools exist to quantify the volumes of the medial temporal lobes, but their accessibility and utilization differ among radiological departments (25). Additionally, the measurement method employed can introduce bias in the determination of absolute hippocampal volumes, as manual volumetry and different automated software programs tend to delineate the anatomical structures in distinct ways (26). In regards to clinical practicality, visual assessment of MTA is still superior to volumetric measuring methods. The utilization of Schelten’s scale has exhibited comparable precision in differentiating between healthy individuals and those afflicted with AD, as automated measurement methods (27, 28).

Studies using MRI and postmortem examinations indicate a link between reduced hippocampal size and depression (29–31), but more research is necessary to determine the significance of hippocampal atrophy in distinguishing LLD from AD. In addition, the Trail Making Test (TMT) holds a prominent position as one of the most frequently employed neuropsychological assessments in clinical practice, particularly within memory clinics (32). Its extensive utilization stems from its historical application in examining impairments pertaining to cognitive processing speed and executive function in individuals (33). Notably, the TMT’s ease of administration and relatively brief duration render it highly suitable for implementation in clinical assessment environments. This study had two primary aims: Firstly, to examine how executive function and MTA differ between stable LLD patients and AD patients; and secondly, to assess how well the TMT and the MTA scale can distinguish between the two disorders.

## Methods

### Participants

A total of 50 patients diagnosed with LLD and 50 with AD were recruited between October 1, 2020, and February 25, 2021, from Xuanwu Hospital, Capital Medical University. LLD patients who were right-handed, between the ages of 60 and 80, and had been taking the same dose of antidepressant medication for over 4 weeks were included. It is noteworthy that within the Diagnostic and Statistical Manual of Mental Disorders-5 (DSM5), hopeless, sadness, and emptiness serve as subjective markers for depressive mood (DSM5, p160) (34). Nevertheless, the precise specificity of “hopeless” as an indicator for major depressive episode remains uncertain in the absence of depressed mood. In clinical settings, this modification has the potential to broaden the diagnostic range of LLD (35), given the prevalence of withdrawal, apathy, and lack of vigor among elderly individuals. Actually, the majority of psychopathological schools posited that there existed a distinction between “hopeless” and “depressed mood” in terms of their phenomenological characteristics (36, 37). Unlike the DSM5, the DSM-IV utilizes subjective markers sadness, and emptiness to identify depression, while excluding marker hopeless. Furthermore, in terms of the agreement in evaluation among clinical practitioners, the DSM-IV demonstrates a higher level of consistency compared to the DSM5 (38). Hence, the DSM-IV criteria was employed in this study. Participants were excluded if they had other major psychiatric disorder, were currently or previously dependent on drugs or alcohol, had a neurological illness such as stroke, transient ischemic attacks, or dementia, were taking medication that prevented cognitive testing, had metal implants that prevented MRI scanning, or had undergone electroconvulsive therapy within the last 6 months. The diagnosis of AD was established using the criteria for clinically probable AD from the National Institute of Neurological and Communicative Diseases and the Stroke-Alzheimer’s Disease and Related Disorders Association, as well as the DSM-IV criteria for Alzheimer’s type dementia. The time span from the clinical diagnosis to enrollment in the study varied from 1 month to 6 years. The institutional review board of Xuanwu Hospital of Capital Medical University examined and sanctioned the study protocols, and all participants furnished written informed consent. In cases where patients are incapable of signing the informed consent form due to cognitive impairment, their guardians will act as their replacements.

### Assessment of depressive symptoms and cognitive function

To evaluate the severity of depressive symptoms and cognitive impairment, we employed various rating scales, such as the Geriatric Depression Scale (GDS) for depression, the Mini-Mental Status Exam (MMSE), and the Montreal Cognitive Assessment (MoCA) for cognition. Tailored for the elderly, the GDS comprises 30 items and adopts a yes-no/agree-disagree response format, simplifying the symptom reporting process for seniors, particularly those with cognitive, physical, or sensory limitations (39). A score between 11 and 20 suggests mild depression, whereas a score of 21 to 30 implies moderate to severe depression. We used the MMSE and MoCA for screening for cognitive impairments. It has been reported that the MMSE is limited in its ability to detect early dementia (40). The MMSE score range for normal elderly individuals overlaps with that of most individuals who meet the clinical criteria of MCI (41). Therefore, we complemented it with the MoCA (42). The MoCA assessment is a 30-point evaluation that spans multiple cognitive areas and can be completed in approximately 10 min on a single page (43). A meta-analysis conducted previously demonstrated that the MoCA exam is a more suitable screening tool for identifying MCI in patients over the age of 60 than the MMSE (44). To evaluate executive function, we employed the TMT, which comprises two sub-tests, namely TMT-A and TMT-B (45). In TMT-A, the participant is instructed to connect circles containing numbers from 1 to 25 in ascending order as fast as they can, which are arranged randomly. To complete TMT-B, the participant must switch back and forth between letters and numbers while moving in a sequential manner. TMT-A measures visual scanning, graphomotor speed, and visuomotor processing speed, while TMT-B assesses working memory and inhibition control through its performance and derived scores (46).

### Image acquisition and visual assessment of MTA

**Figure 1:**
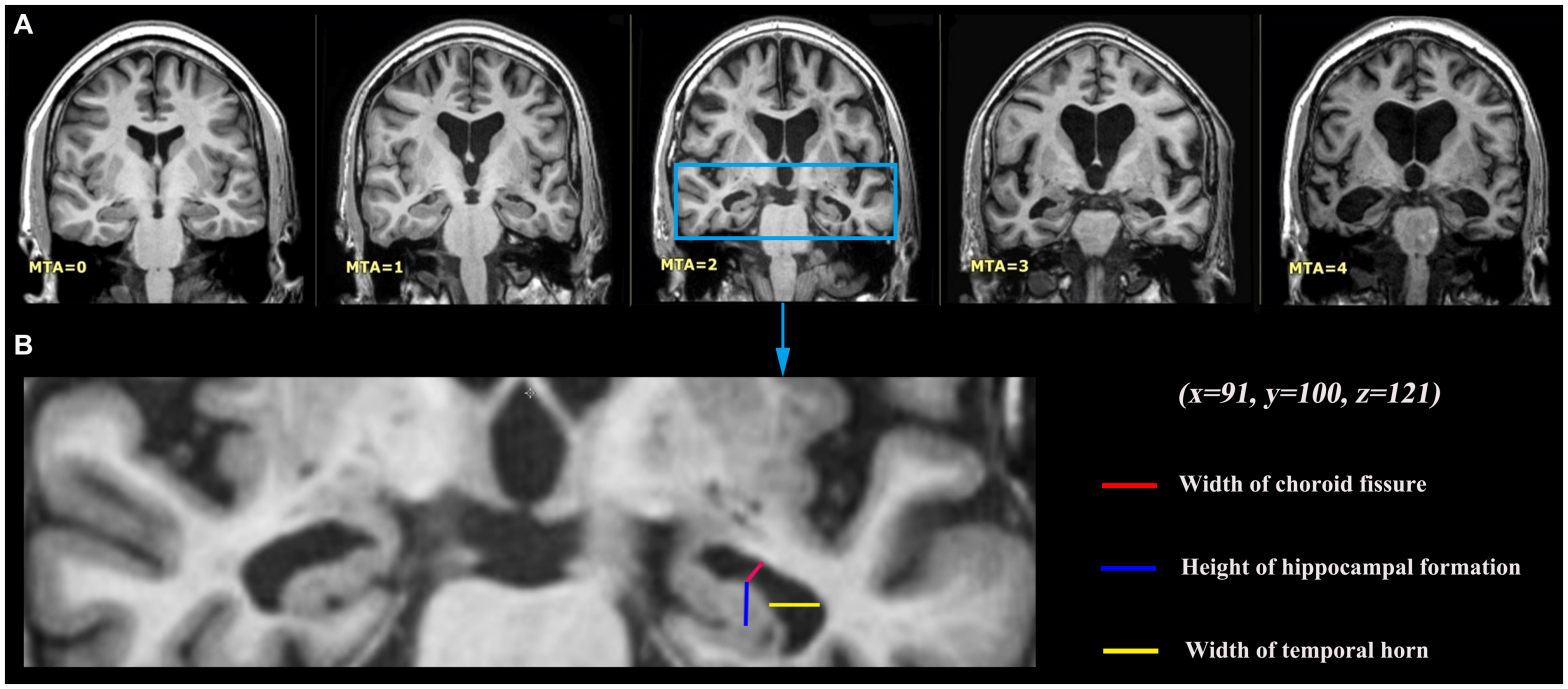
Medial temporal lobe atrophy (MTA) grading criteria. **(A)** Coronal T1-weighted slices at the level of the hippocampus body of five different study participants with different levels of MTA, 0 no atrophy; 1 widening of the choroid fissure; 2 additional widening of the temporal horn of the lateral ventricle and slightly decreased hippocampal height; 3 moderate loss of hippocampal volume; 4 end-stage increase of all these findings. Those were presented by MRIcron software, with the same coordinate (x = 91, y = 100, z = 121). **(B)** MTA visual rating is performed on a single coronal slice including hippocampus, with particular attention to the width of the choroid fissure and of the temporal horn, and to the hippocampus height.

To obtain high-resolution 3D T1-weighted images of the brain of each participant, we utilized the Siemens 3 T scanner (Siemens, Erlangen, Germany) and performed a turbo field echo (3D TFE) scan. 182 axial slices were scanned with a repetition time (TR) of 9.6 ms, an echo time (TE) of 4.6 ms, a flip angle of 8 degrees, a slice thickness of 1.2 mm, and an in-plane voxel size of 0.98 × 0.98 × 1.2 mm^3^. The participants’ heads were secured with foam pads and straps to minimize motion. According to Scheltens’ original description (27), the MTA scale was utilized to rate 3D T1-weighted MR images. Specifically, the rating was conducted on the coronal slice that was presented by MRIcron solfware in the Nii format. During this assessment, both hemispheres were evaluated and assigned a score ranging from 0 to 4 based on the following criteria: 0 indicating no atrophy, 1 indicating widening of the choroid fissure, 2 indicating additional widening of the temporal horn of the lateral ventricle and a slight decrease in hippocampal height, 3 indicating moderate loss of hippocampal volume, and 4 indicating end-stage increase of all aforementioned findings (Figure 1). The MTA rating was conducted by two proficient neuro-radiologists, who provided ratings and feedback for a total of 100 data sets. In instances where there was disagreement between the two raters regarding the highest score in each scale for the two hemispheres, the raters engaged in collaborative discussions to achieve a final consensus. Due to the minimal discrepancy of one point observed in the majority of discordant scores, primarily resulting from evaluations conducted on neighboring slices by the raters, the final consensus was often readily achieved by selecting the slice exhibiting more pronounced atrophy.

### Statistical analysis

SPSS 26.0 (SPSS, Chicago, IL, United States) was used for statistical analysis. Regression analysis utilizes independent descriptive factors to forecast the outcome variable. The mean ± SD was used to express age, while frequencies and percentages were used to report categorical variables such as gender. Counts and percentages were used to express sex. Continuous variables were compared using an independent *t*-test, while categorical variables were compared using either a chi-square test or a Mann–Whitney U test. Statistically significant results were defined as having *p*-values less than 0.05. To assess the ability of TMT and MTA grading to distinguish between the two groups, we created a receiver operating characteristic (ROC) curve. The correlations of the MTA grading with neuropsychologic deficits and depressive symptoms were assessed using Pearson or Spearman correlation analysis.

## Results

### Comparison of demographics, neuropsychology and MTA grading between the two groups

Table 1 displays the traits of the individuals involved. The mean ages of the patients with AD and LLD were 71.20 ± 6.38 and 70.04 ± 4.62, respectively. Both groups had similar age, gender, and educational levels. Notably, the two groups displayed noteworthy disparities in illness course, GDS, MMSE, MoCA, TMT-A, TMT-B, and MTA total, with a significance level of *p* < 0.05.

**Table 1:** Comparison of demographics, neuropsychology and MTA grading between 50 AD and 50 LLD patients.

Characteristic	AD group	LLD group	Statistics	*p* value
	(*n* = 50)	(*n* = 50)	t/x^2^	
Sex (male/female)	14/36	13/37	0.051	0.822
Age (years)	71.20 ± 6.38	70.04 ± 4.62	1.041	0.300
Education (years)	8.73 ± 3.67	10.16 ± 3.65	−1.955	0.053
Illness Course (years)	3.84 ± 1.39	1.69 ± 1.61	10.004	< 0.001
GDS (score)	16.38 ± 5.05	20.00 ± 3.71	−4.085	< 0.001
MMSE (score)	15.78 ± 5.08	24.30 ± 2.22	−10.86	< 0.001
MoCA (score)	11.42 ± 3.81	18.60 ± 3.87	−9.352	< 0.001
TMT-A (s)	128.27 ± 49.85	67.92 ± 25.54	8.880	< 0.001
TMT-B (s)	270.60 ± 64.07	103.70 ± 57.93	13.663	< 0.001
MTA total (score)	3.74 ± 1.38	2.30 ± 1.16	5.633	< 0.001

AD, Alzheimer’s disease; LLD, late-life depression; GDS, Geriatric Depression Scale; MMSE, Mini-mental State Examination; MoCA, Montreal Cognitive Assessment; TMT-A, trail making test part A; TMT-B, trail making test part B; MTA, medial temporal lobe atrophy.

### ROC curve analysis

**Figure 2:**
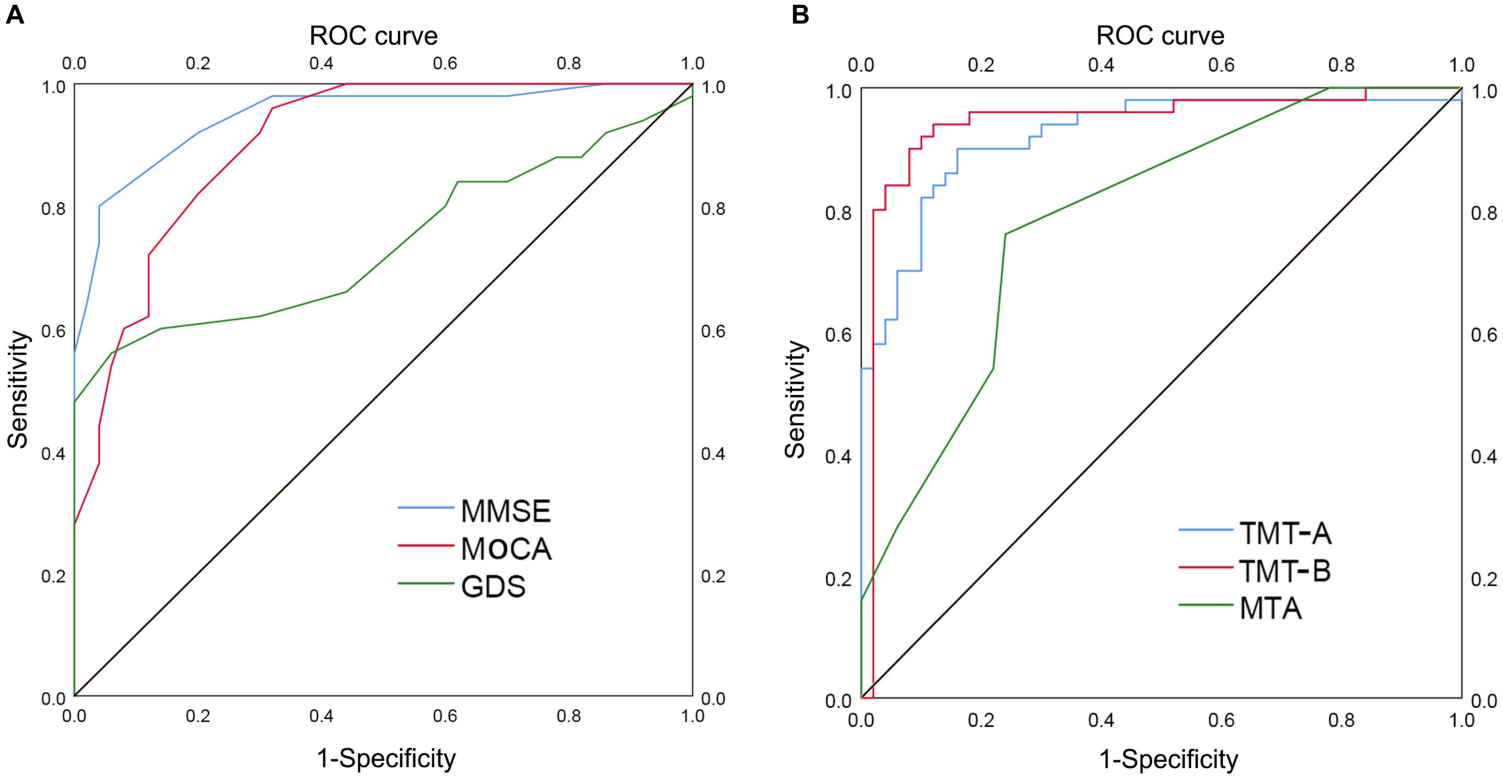
ROC curve analysis of TMT and MTA grading in distinguishing AD group from LLD group. The utilization of ROC curve analysis was employed to assess the discriminatory capacity of the TMT test and MTA grading in distinguishing between the two groups. A higher value of the AUC signifies enhanced diagnostic accuracy. **(A)** AUC of MMSE (0.948), MoCA (0.904), and GDS (0.738) in distinguishing AD group from LLD group, respectively. **(B)** AUC of TMT-A (0.922), TMT-B (0.942), and MTA (0.787) in distinguishing AD group from LLD group, respectively. ROC, receiver operating characteristic; AUC, area under curve; TMT, Trail Making Test; MTA, medial temporal lobe atrophy; MMSE, Mini-Mental State Examination; MoCA, Montreal Cognitive Assessment; GDS, Geriatric Depression Scale; AD, Alzheimer’s disease; LLD, late-life depression.

To evaluate the ability of TMT test and MTA grading to distinguish between the two groups, we employed ROC curve analysis and other diagnostic techniques. Improved diagnostic accuracy is indicated by a greater value of the area under the curve (AUC). Figure 2A and Table 2 showed that the AUC values for MMSE, MoCA, and GDS tests were 0.948 (*p* < 0.001; 95% CI 0.906–0.990, sensitivity 0.800, specificity 0.960), 0.904 (*p* < 0.001; 95% CI 0.847–0.961, sensitivity 0.960, specificity 0.680), and 0.738 (*p* < 0.001; 95% CI 0.636–0.840, sensitivity 0.560, specificity 0.940), respectively. Figure 2B and Table 2 showed that TMT-A, TMT-B, and MTA grading had AUC values of 0.922 (*p* < 0.001; 95% CI 0.867–0.976, sensitivity 0.900, specificity 0.840), 0.942 (*p* < 0.001; 95% CI 0.888–0.997, sensitivity 0.900, specificity 0.920), and 0.787 (*p* < 0.001; 95% CI 0.698–0.876, sensitivity 0.760, specificity 0.760), respectively, indicating a contrast in their performance.

**Table 2:** ROC curve analysis of MMSE, MoCA, GDS, TMT-A, TMT-B, and MTA total score in distinguishing AD patients from LLD patients.

Variables	AUC, 95%CI	Sensitivity, %	Specificity, %	Cut off point
MMSE	0.948 (0.906–0.990)	0.800	0.960	20.5
MoCA	0.904 (0.847–0.961)	0.960	0.680	17.5
GDS	0.738 (0.636–0.840)	0.560	0.940	15.5
TMT-A	0.922 (0.867–0.976)	0.900	0.840	78.91
TMT-B	0.942 (0.888–0.997)	0.900	0.920	176.71
MTA score total	0.787 (0.698–0.876)	0.760	0.760	2.500

**Figure 3:**
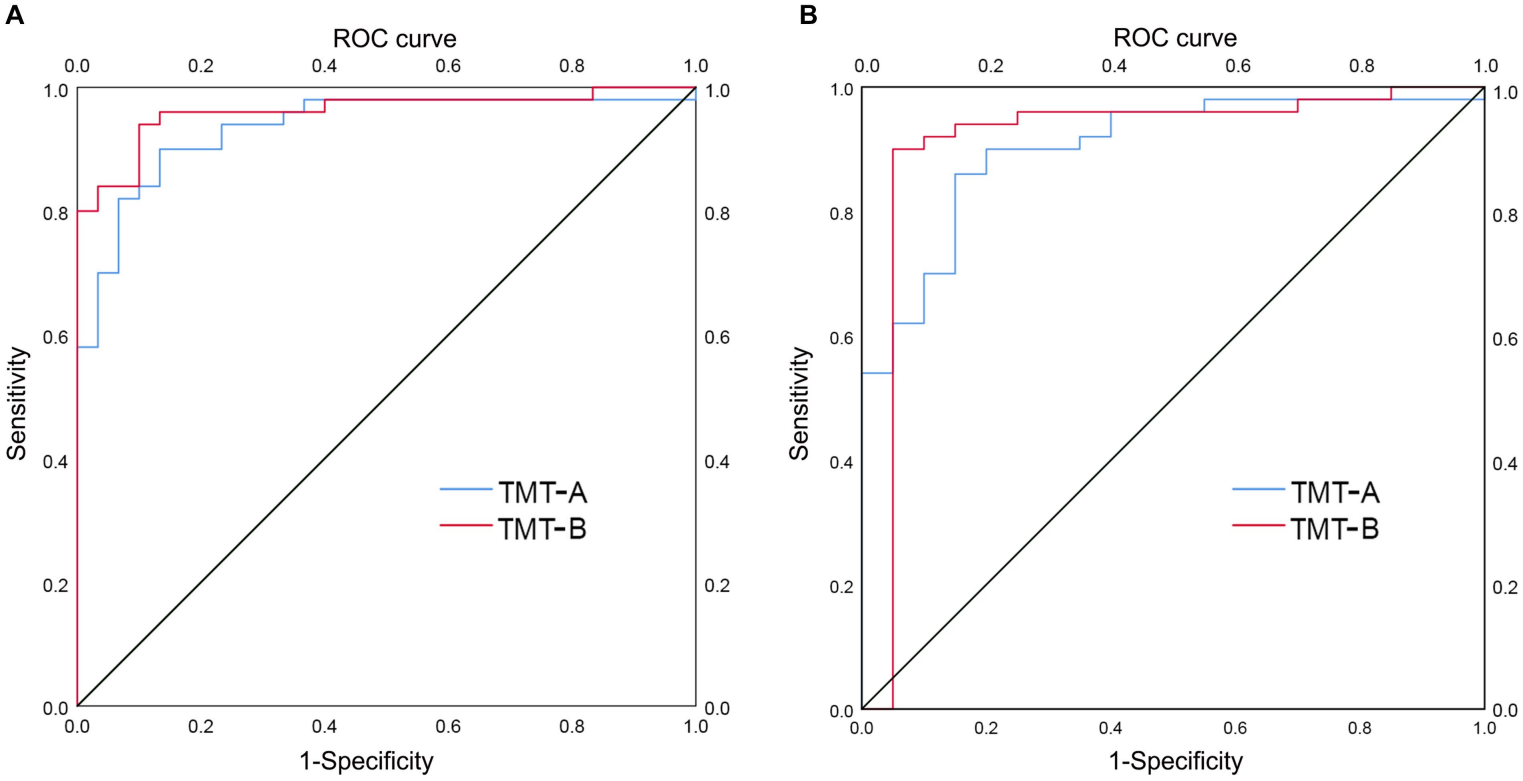
ROC curve analysis of TMT-A and TMT-B in distinguishing AD group from LLD group at various stages. **(A)** AUC of TMT-A (0.935) and TMT-B (0.961) in distinguishing AD group (*n* = 50) from group with mild LLD (*n* = 30), respectively. **(B)** AUC of TMT-A (0.902) and TMT-B (0.914) in distinguishing AD group (*n* = 50) from group with moderate to severe LLD (*n* = 20), respectively.

A subgroup analysis was performed to detect the ability of TMT-A and TMT-B to distinguish AD from LLD at various stages. In patients with AD (*n* = 50) and mild LLD (n = 30), TMT-A and TMT-B had AUC values of 0.935 (*p* < 0.001; 95% CI 0.880–0.990, sensitivity 0.900, specificity 0.867) and 0.961 (*p* < 0.001; 95% CI 0.921–1.000, sensitivity 0.940, specificity 0.900), respectively (Figure 3A). In patients with AD (n = 50) and moderate to severe LLD (*n* = 20), the AUC values for TMT-A and TMT-B were 0.902 (*p* < 0.001; 95% CI 0.827–0.977, sensitivity 0.860, specificity 0.850) and 0.914 (*p* < 0.001; 95% CI 0.813–1.000, sensitivity 0.900, specificity 0.95), respectively (Figure 3B and Table 3).

**Table 3:** ROC curve analysis of TMT-A and TMT-B in distinguishing AD patients from LLD patients at various stages.

Variables	Patients with AD or mild LLD				Patients with AD or moderate to severe LLD			
	AUC, 95%CI	Sensitivity, %	Specificity, %	Cut off point	AUC, 95%CI	Sensitivity, %	Specificity, %	Cut off point
TMT-A	0.935 (0.880–0.990)	0.900	0.867	78.74	0.902 (0.827–0.977)	0.860	0.850	80.71
TMT-B	0.961 (0.921–1.000)	0.940	0.900	148.53	0.914 (0.813–1.000)	0.900	0.950	176.71

### Correlation analysis

**Figure 4:**
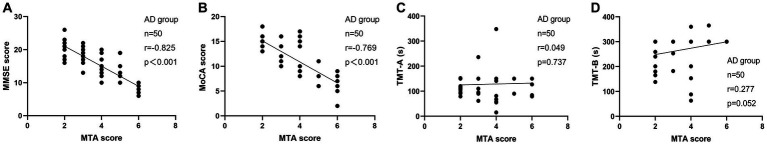
Correlation analysis between MTA score and neuropsychologic deficits in patients with AD. **(A)** The correlation between MTA score with MMSE score; **(B)** The correlation between MTA score with MoCA score; **(C)** The correlation between MTA score with TMT-A time spent; **(D)** The correlation between MTA score with TMT-B time spent.

**Figure 5:**
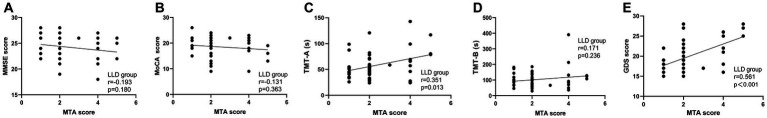
Correlation analysis between MTA score and neuropsychologic deficits and depressive symptoms in patients with LLD. **(A)** The correlation between MTA score with MMSE score; **(B)** The correlation between MTA score with MoCA score; **(C)** The correlation between MTA score with TMT-A time spent; **(D)** The correlation between MTA score with TMT-B time spent; **(E)** The correlation between MTA score with GDS score.

We examined the correlations between MTA score and MMSE, MoCA, TMT-A, and TMT-B in the AD group. Similarly, we also investigated the associations between MTA score and MMSE, MoCA, TMT-A, TMT-B, and GDS in the LLD group (Figures 4, 5). Among AD patients, there was an inverse relationship observed between the MTA score and both the MMSE score (*r* = −0.825, *p* < 0.001, *n* = 50, Figure 4A) and the MoCA score (*r* = −0.769, *p* < 0.001, *n* = 50, Figure 4B). A correlation was observed in the LLD group between the MTA score and TMT-A score (*r* = 0.351, *p* = 0.013, *n* = 50, Figure 5C) and GDS score (*r* = 0.561, *p* < 0.001, *n* = 50, Figure 5E), respectively.

## Discussion

The purpose of this research was to investigate how TMT and MTA can aid in distinguishing between AD and LLD. Our findings indicate that individuals with AD exhibit greater cognitive decline, particularly in executive function, and more pronounced MTA compared to those with LLD. We also compared the MMSE and MoCA, two commonly used screening tools for cognitive measurement, with the simpler TMT, and we found similar discriminatory power. In LLD patients, we noticed a positive association between MTA classification and TMT-A and GDS, but this was not observed in the AD group.

Numerous studies have demonstrated that MoCA is more effective than MMSE in recognizing MCI due to the absence of a ceiling effect, and both assessments are precise in detecting AD (47–49). Our research also revealed that MMSE had a lesser ability to differentiate AD from LLD than MoCA (0.80% vs. 0.96%), despite its AUC being marginally greater than that of MoCA (94.8% vs. 90.4%). Studies that follow patients over time have shown that the TMT test can forecast changes in clinical and functional abilities for those with AD, LLD, and MCI (16, 50, 51). Nevertheless, there are limited studies that have employed the TMT to differentiate between AD and LLD. The A and B subtests both had an AUC of over 90%, indicating excellent performance by TMT. The clinical potential of TMT-B is excellent due to its sensitivity and specificity, which are above 90%.

As per a recommended meta-analysis, LLD could be an early sign of AD (52). In comparison to AD patients, individuals with LLD are usually less affected by their everyday activities. The TMT-B evaluates cognitive flexibility and working memory in elderly individuals, which are crucial for everyday activities such as dressing and cooking. Difficulty in daily living is often experienced by older individuals with inadequate TMT-B performance (53), indicating that TMT-B could be a promising approach to differentiate between LLD and AD. The impact of depression severity on the cognitive functioning of individuals with LLD, particularly during severe depressive episodes, is evidently significant. In order to investigate the distinction between AD and LLD at various stages, a subgroup analysis was performed, focusing on the TMT-A and TMT-B. Our findings indicated that regardless of whether LLD is mild or moderate to severe, TMT-B exhibited an AUC area, sensitivity, and specificity exceeding 90%, thereby reinforcing the validity of our finding. However, some participants did not finish TMT-B within the required time, and we used the maximum time allowed to complete the test in the statistical analysis following the operational manual, which might have introduced some bias.

AD is closely related to brain atrophy, particularly in the hippocampus or MTA. In the early stages of AD (54), neurofibrillary tangles emerge in the medial temporal lobe, beginning in the transentorhinal cortex and entorhinal cortex before spreading to the hippocampal subfields and eventually reaching isocortical areas in the late stages of the disease. Despite numerous studies investigating the hippocampus in LLD (55), few have examined its subfields and associated medial temporal lobe regions (56). Our study discovered that the severity of MTA was greater in AD patients than in LLD patients, as anticipated. According to a recent meta-analysis (57), MTA has the potential to be a promising biomarker for AD progression. Additionally, we observed a negative correlation between MTA and cognitive impairment in the AD group. Nonetheless, the MTA assessment did not perform as well as the TMT in distinguishing between the two groups of patients.

Depression-executive dysfunction syndrome is a term used to describe a unique clinical presentation and inadequate response to antidepressants that may be observed in a certain group of elderly individuals with depression (58). The condition is linked to deficiencies in multiple managerial abilities, including but not limited to speaking proficiency, restraining responses, devising innovative solutions, adapting to new situations, retaining information, and/or planning physical actions (59, 60). In this investigation, we observed a favorable association between MTA and the duration of TMT-A. In LLD research, the hippocampus has been thoroughly examined, and a meta-analysis of 35 studies found that patients with LLD had significantly smaller hippocampal volumes compared to those without the condition. In LLD patients, cognitive impairment, particularly in semantic retrieval and verbal memory, is strongly associated with hippocampal atrophy (61, 62).

Despite the absence of a statistically significant distinction in age and education between the two groups in this study, it became apparent that the mean age of individuals in the AD group exceeded that of the LLD group in numerical terms, while the years of education exhibited an inverse relationship. Taylor et al. discovered that, increasing age had a greater negative correlation with medial temporal volumes in the depressed group than in the nondepressed group (mean age = 66.4 years, SD = 5.8y, range 60-86y) (56). Moreover, Tang et al. conducted a study that demonstrated a positively association between education and size variations in the CA1 and subiculum subregions of the hippocampus in a sample of non-dementia elderly individuals (63). Prior research has indicated that education plays a crucial role in enhancing cognitive resilience, which could potentially influence the outcomes of cognitive assessments, such as the MoCA and the MMSE (64, 65). Consequently, when differentiating between LLD and AD in clinical settings, it is imperative to thoroughly consider the potential confounding effects of age and education.

In addition to neurocognition and MRI, a limited number of studies have explored alternative biomarkers, including electroencephalography (EEG) and cerebrospinal fluid (CSF) measurement. A previous investigation demonstrated that the utilization of EEG with quantitative analysis method can effectively differentiate between patients with LLD and those with AD, exhibiting a sensitivity and specificity of approximately 92% (66). Furthermore, Liguori et al. have substantiated the efficacy of CSF measurement, as they have observed elevated levels of CSF tau proteins and diminished concentrations of CSF Aβ42 in individuals with AD compared to both those with LLD and control subjects (67). These findings can be utilized to discriminate between the two conditions. As a whole, the existing tools or approaches suggested for early differentiation between LLD and AD remain arduous and lack unanimity.

The MTA score showed a positive correlation with GDS in the LLD group but not in the AD group. MTA may indicate neuroendocrine alterations in the HPA axis, which plays a role in managing stress and mood in patients with LLD. Stress usually triggers the activation of the hypothalamic–pituitary–adrenal (HPA) axis, causing the hypothalamus to release corticotropin-releasing factor (CRF), which then stimulates the pituitary gland to secrete adrenocorticotropic hormone (ACTH) (68). ACTH then triggers the release of glucocorticoids (GCs) from the adrenal glands into the bloodstream. Physiological adaptations in response to stress are made possible by GCs, as well as epinephrine and norepinephrine from the sympathetic nervous system (69). However, in both AD and LLD patients, the HPA axis is dysregulated, leading to impaired negative feedback and increased GCs levels in the brain (70, 71). This results in reduced volumes of the hippocampal and PFC cues (72), as well as increased vulnerability of neurons to free oxygen radicals and Aβ, which are common features of both AD and LLD (73). Mood disorders and cognitive impairment, particularly EFD, have a strong correlation with these modifications.

It is important to recognize some constraints in this research. Due to the constraints of clinical diagnosis and the absence of follow-up information, it was impossible to exclude the likelihood that certain LLD patients were preclinical AD cases with depressive symptoms prior to the onset of cognitive impairments. Additionally, our findings may be limited in their generalizability and causal inference due to the relatively small sample size and cross-sectional design. Thirdly, manual evaluation of MTA was utilized, which may not be practical in typical clinical environments. Fourth, the absence of AD staging hindered the possibility of conducting subgroup analysis to delve deeper into the correlation between MTA and different stages of AD. Fifth, we did not count for potential confounders such as antidepressant use or vascular alterations in the brain that could directly or indirectly affect hippocampus volume. Sixth, the omission of AD pathological biomarkers, such as CSF tau/Aβ or amyloid-PET, which are currently integral to the *in vivo* diagnosis of AD, is a noteworthy aspect that warrants attention in future research. Finally, we only measured the time spent on the executive functional tests (TMT-A or TMT-B), and we did not include other indicators such as error rate pen lift reminders.

## Conclusion

To summarize, our study of patients with LLD or AD using standard cognitive assessment tools and tests for executive function revealed that AD patients had more severe EFD and MTA compared to those with LLD. Moreover, TMT exhibited encouraging capability for clinical application in differentiating AD from LLD. It is important to acknowledge that TMT is capable of capturing only a fraction of the executive function, thus necessitating a cautious interpretation of research findings.

## Data availability statement

The raw data supporting the conclusions of this article will be made available by the authors, without undue reservation.

## Ethics statement

The studies involving humans were approved by The institutional review board of Xuanwu Hospital of Capital Medical University. The studies were conducted in accordance with the local legislation and institutional requirements. The participants provided their written informed consent to participate in this study.

## Author contributions

The research protocol was conceptualized and designed by CC, while PM and WP were responsible for conducting the data analyses. CY provided assistance with neuropsychological assessment and data processing, while YR and CY verified the MRI data. The manuscript underwent revision and language refinement by CL and Y-lT. All authors made contributions to the article and gave their approval for the submitted version.

## Funding

The present work was supported by the National Key Research and Development Program of China (No. 2022YFC3602605) and Beijing Municipal Science & Technology Commission (No. Z191100006619105).

## Conflict of interest

The authors declare that the research was conducted in the absence of any commercial or financial relationships that could be construed as a potential conflict of interest.

## Publisher’s note

All claims expressed in this article are solely those of the authors and do not necessarily represent those of their affiliated organizations, or those of the publisher, the editors and the reviewers. Any product that may be evaluated in this article, or claim that may be made by its manufacturer, is not guaranteed or endorsed by the publisher.
